# Tonic Pupil Following Pars Plana Vitrectomy and Endolaser

**DOI:** 10.1155/2009/970502

**Published:** 2009-07-08

**Authors:** Benyamin Ebrahim, Larry Frohman, Marco Zarbin, Neelakshi Bhagat

**Affiliations:** ^1^The Institute of Ophthalmology and Visual Science, New Jersey Medical School (NJMS), Newark, USA; ^2^Department of Neurology and Neurosciences, New Jersey Medical School (NJMS), Newark, USA

## Abstract

Tonic pupil was observed in a 67 year-old patient following a retinal detachment repair with pars plana vitrectomy, endolaser and silicone oil tamponade performed under retrobulbar anesthesia. The probable location of disturbance is the postganglionic parasympathetic fibers in the short ciliary nerves along their course to the pupil in the suprachoroidal space. A likely explanation for this phenomenon is injury to short ciliary nerves by endolaser treatment.

## 1. Introduction

The tonic pupil is a dilated pupil with poor constriction to light and usually with a slow constriction response to accommodation, often with tonic redilation and segmental denervation of the iris sphincter. In the prepresbyopic patient, loss of accommodation may be a prominent feature. This phenomenon has been termed pupillotonia, tonic pupil, Adie's pupil, and myotonic pupil. When these abnormalities are bilateral and accompanied by abnormal or absent deep tendon reflexes, the condition usually is referred to as Adie's tonic pupil syndrome [1]. 

Idiopathic tonic pupil has a predilection for females and is most common in the third to fifth decades of life. Generally, it is a unilateral condition, but bilateral cases have been reported [1, 2]. While tonic pupil is most often idiopathic, trauma to the eye remains the most common known cause. The diagnosis of tonic pupil typically is confirmed by the demonstration of denervation supersensitivity to dilute pilocarpine, usually 0.1% [3, 4]. The tonic pupil generally is benign and usually remains stable without progression. Occasionally minimal improvement can be noted, and the size of the tonic pupil can decrease over time [1].

There is limited published literature on this phenomenon following retinal detachment operations. It is possible that tonic pupils are overlooked in postoperative patients who often are examined after their pupils have been dilated [4]. The occurrence of tonic pupil after retinal detachment repair with scleral buckle has been described by Adler and Scheie [2], and Newsome and Einaugler [5]. Tonic pupil had been considered as a possible postoperative complication of retinal detachment procedures, particularly scleral buckle and diathermy [2, 5]. 

## 2. Methods

### 2.1. Case

A 67 year-old male patient, with medical history significant for chronic lymphocytic leukemia (CLL) and coronary artery disease, and ocular history significant for cataract extraction and posterior chamber intraocular lens placement (CE/PC/OL) in both eyes two years previously, was treated for a macula-involving rhegmatogenous retinal detachment in the right eye. The retinal detachment extended from 3 to 10 o’clock hours with a retinal flap tear at 8 o’clock just anterior to the equator. The preoperative visual acuity in the affected eye was 20/200. The pupils were equal, round, and reactive to light. The patient underwent pars plana vitrectomy (PPV), endolaser (EL), and silicone oil (SO) tamponade in the right eye under local anesthesia (surgeon NB). Retrobulbar anesthesia was performed with 5 cc of a 50 : 50 mixture of lidocaine 2% and marcaine 0.5%. The patient was pseudophakic with an intact lens capsule. No iridectomy was performed. He was placed on atropine postoperatively for 6 weeks. However, 4 weeks after discontinuation of atropine, the patient still had a fully dilated right pupil.

## 3. Results

Fourteen weeks after surgery, the patient, no longer on any ocular medications, continued to have a mid-dilated right pupil with no reverse afferent pupillary defect (RAPD). The corrected visual acuity was 20/60 in the right eye and 20/20 in the left. The pupil size was 4.5 mm on the right and 1.8 mm on the left. The right pupil did not react to light, however, as the right eye was illuminated, the left pupil was seen to constrict via the consensual response. The right eye constricted slightly to near. The response to dilute pilocarpine 0.1% confirmed the presence of denervation supersensitivity in the right eye and the diagnosis of tonic pupil.

## 4. Discussion

The muscles responsible for the pupillary light reflex and the accommodation reflex (the iris sphincter and the ciliary muscle) are innervated by postganglionic parasympathetic fibers in the short ciliary nerves. The preganglionic parasympathetic fibers initiate their course in the Edinger-Westphal nucleus in the midbrain and then run in the inferior division of the oculomotor nerve until they synapse in the posterior orbit at the ciliary ganglion [1, 6]. The ciliary ganglion, which is located behind the globe between the optic nerve and the lateral rectus in the posterior region of the orbit, gives rise to the short ciliary nerves, which carry the post-synaptic parasympathetic fibers [6]. These parasympathetic nerves travel through the sclera in the territory of the optic nerve and continue their course in the suprachoroidal space forward to innervate the iris sphincter and the ciliary muscle [4, 6]. The likely anatomic lesion that causes a tonic pupil is an injury to the postganglionic parasympathetic fibers along their course from the ciliary ganglion to the pupillary and the ciliary muscles [2, 4, 5]. 

In the case we presented above, the patient had a half an hour retinal flap tear at 8 o’clock position anterior to the equator; endolaser was performed around the tear in 3 rows using argon laser (Power 300 mW, duration 0.2 seconds, wavelength 532 nm). Therefore, it is possible that the endolaser may have induced injury of the short ciliary nerves along their course in the orbit to the sphincter muscle. Pupillary abnormalities have been described in other ocular surgeries. Atonic (but not tonic) pupil has been reported as a complication of other ocular surgeries [7, 8]. Atonic pupil is an enlarged, irregular, poorly reactive pupil resulting from dysfunction of the iris sphincter. Eight cases of atonic pupil, all of which were men between the ages of 33 to 74 at the time of surgery, have been reported following cataract surgery performed both under general anesthesia [7] as well as with retrobulbar/peribulbar anesthesia [8]. Six of the reported cases had intracapsular cataract extraction with anterior chamber IOL implantation, and the other two cases had extracapsular cataract extraction, one with posterior chamber IOL implantation and the other without implantation. Except for one patient with 20-diopter myopia and another with primary open angle glaucoma, the patients had no previous ocular history. These pupils did not show denervation supersensitivity. In these cases, mechanical and/or toxic injury to the iris sphincter was postulated as a possible mechanism [7, 8].

This report describes development of a tonic pupil after PPV and endolaser. To the best of our knowledge, no cases of tonic pupil following PPV have been reported in the literature. One case of tonic pupil has been described after scleral buckle and diathermy for retinal detachment repair [5]. In our case, neither scleral buckle, diathermy, nor cryopexy were performed. Endolaser was performed around the retinal tear at 8 o’clock.

We speculate that the tonic pupil in our patient was caused by endolaser-induced damage to the short ciliary nerves in the suprachoroidal space. Thermal damage to the short ciliary nerves as they run anteriorly in the choroid or the suprachoroidal space can occur with laser photocoagulation [2, 4]. Four cases of tonic pupil with denervation supersensitivity have been described by Patel et al*. * following diode laser photocoagulation with subtenon local anesthetic [4]. These patients were treated with diode laser, but no incisional surgery was performed. The authors hypothesize the site of the injury was most likely the parasympathetic fibers in the short ciliary nerves as they pass through the suprachoroidal space [4].

Retrobulbar local anesthetic injection can potentially lead to increased intra orbital pressure in the infratemporal region, where the ciliary body is located, thereby causing injury to the short ciliary nerves carrying the postganglionic parasympathetic fibers [4, 9], possibly leading to a tonic pupil. Direct needle injury to the ciliary ganglion and/or the short ciliary nerves is also possible. However, given the wide use of subtenon and retrobulbar anesthesia, and the lack of tonic pupil cases reported after these procedures, it seems unlikely that retrobulbar anesthesia is the sole underlying mechanism for the tonic pupil in our case. It is possible, however, that there is an additive effect between the retrobulbar anesthesia and the endolaser therapy. The tonic pupil, in this case, can be idiopathic. The prevalence of idiopathic tonic pupil is approximately two cases per thousand. The mean age of onset is around 32 years with a female predominance [10].

## 5. Conclusion

A case of tonic pupil following PPV and endolaser with retrobulbar anesthesia has been described. The probable location of disturbance is the postganglionic parasympathetic fibers along their course in the suprachoroidal space. A likely explanation for this phenomenon is injury to the short ciliary nerves by endolaser treatment. Further investigative studies are needed to illustrate the role of PPV, endolaser, and retrobulbar anesthesia in inducing tonic pupil syndrome.
